# Comparative Genomic Analysis Reveals Organization, Function and Evolution of *ars* Genes in *Pantoea* spp.

**DOI:** 10.3389/fmicb.2017.00471

**Published:** 2017-03-21

**Authors:** Liying Wang, Jin Wang, Chuanyong Jing

**Affiliations:** ^1^State Key Laboratory of Environmental Chemistry and Ecotoxicology, Research Center for Eco-Environmental Sciences, Chinese Academy of SciencesBeijing, China; ^2^College of Resources and Environment, University of Chinese Academy of SciencesBeijing, China; ^3^Department of Municipal and Environmental Engineering, School of Civil Engineering, Beijing Jiaotong UniversityBeijing, China

**Keywords:** comparative genomic, arsenic, *Pantoea* spp., arsenic resistance, *ars* genes

## Abstract

Numerous genes are involved in various strategies to resist toxic arsenic (As). However, the As resistance strategy in genus *Pantoea* is poorly understood. In this study, a comparative genome analysis of 23 *Pantoea* genomes was conducted. Two vertical genetic *arsC*-like genes without any contribution to As resistance were found to exist in the 23 *Pantoea* strains. Besides the two *arsC*-like genes, As resistance gene clusters *arsRBC* or *arsRBCH* were found in 15 *Pantoea* genomes. These *ars* clusters were found to be acquired by horizontal gene transfer (HGT) from sources related to *Franconibacter helveticus, Serratia marcescens*, and *Citrobacter freundii*. During the history of evolution, the *ars* clusters were acquired more than once in some species, and were lost in some strains, producing strains without As resistance capability. This study revealed the organization, distribution and the complex evolutionary history of As resistance genes in *Pantoea* spp.. The insights gained in this study improved our understanding on the As resistance strategy of *Pantoea* spp. and its roles in the biogeochemical cycling of As.

## Introduction

Arsenic (As), one of the earliest known toxic elements, occurs naturally worldwide (Smith et al., 2002). To adapt to habitats with elevated As, microbes have evolved dynamic resistance mechanisms. The most ubiquitous and important strategy of As resistance is to reduce As(V) to As(III) and extrude it using *ars* operons with various genomic configurations in specific bacterial strains (Páezespino et al., 2009). The core genes of *ars* systems, however, are *arsR* (encoding the transcriptional repressor ArsR), *arsB* (encoding the arsenite efflux pump ArsB) and *arsC* (encoding arsenate reductase ArsC) (Xu et al., 1998). Besides this detoxification mechanism using *ars* systems, some strains possess the mechanism of As methylation-demethylation, changing inorganic As into organic forms using a distinct gene *arsM* (Qin et al., 2006; Zhao et al., 2015). Some strains are able to oxidize As(III) to As(V), which involve membrane-associated proteins, AoxAB (Levin and Tal, 2003; Ghosh et al., 2014). Some strains are able to reduce As(V) to As(III) with ArrAB as part of their respiratory processes transferring electrons to As and producing the energy for strains (Saltikov and Newman, 2003). The reported genes related to the strategy of As resistance are listed in **Table 1**.

**Table 1:** Genes involved in arsenic resistance and transformation.

Function	Gene	Protein
**Arsenate reduction**	*arsR*	Transcriptional regulator ArsR
	*arsB*	As(III) efflux pump protein
	*arsC*	As(V) reductase ArsC
	*arsH*	Putative flavoprotein
	*arsA*	As(III) active ATPase
	*arsD*	As metallochaperone
	*arsO*	Monooxygenase
	*arsT*	Putative thioredoxin system
	*arsX*	Unknown function
	*arsN*	Acetyltransferases
**Respiratory reduction**	*arrA*	Large subunit of respiratory As(V) reductase
	*arrB*	Small subunit of respiratory As(V) reductase
**Arsenite oxidation**	*aioA*	Large subunit of As oxidase
	*aioB*	Small subunit of As oxidase
	*aioD*	Biosynthesis protein A with molybdenum cofactor
	*aioX*	Phosphonate-binding periplasmic protein
	*aioS*	Histidine kinase for signal transduction
	*aioC*	Cytochrome c
**Arsenic methylation**	*arsM*	As(III) S-adenosylmethyltransferase

In traditional molecular biology research, As resistance traits are revealed primarily based on the cultivation of a specific strain, and it is impossible to study the As strategy of all strains in a genus. Nevertheless, understanding of such traits in all strains of a genus is sometime more desirable. Gaining this knowledge is no longer a challenge with the explosive development of high-throughput sequencing technology. The genomic sequence of a strain contains nearly all of the genetic information. Therefore, fundamental knowledge such as the phylogenetic, the genetic traits of As resistance and its evolutionary history can be obtained through comparative genomic analysis (Arsène-Ploetze et al., 2010; Colston et al., 2014). So here we use genomic information of *Pantoea* spp. and compared these genomes to explore and predict the strategy of As resistance and their evolutionary patterns in genus *Pantoea* as an example.

*Pantoea* is a genus of Gram-negative, facultative anaerobic bacteria. This genus belongs to gamma *Proteobacteria*, family *Gammaproteobacteria*, and was recently separated from the genus *Enterobacter* (Gavini et al., 1989). Currently, the genus contains 26 species^1^. Members of this genus are found in various environmental matrices (Meng et al., 1995; Zhang and Birch, 1997; Rezzonico et al., 2009). In 2013, the strain *Pantoea* sp. IMH was an isolate that reported firstly as the strain having the As resistance capability within *Pantoea* species (Wu et al., 2013). Further, we sequenced the genome of *Pantoea* sp. IMH and found two *ars* clusters (*arsR1B1C1H1* and *arsR2B2C2H2*) co-contributing to its As resistance (Tian and Jing, 2014; Wang et al., 2016). However, the evolutionary history and genetic traits of As resistance in genus *Pantoea* are not fully understood.

Herein, we present the first study of the genetic traits of As resistance in *Pantoea* spp., as well as their evolutionary history. Two vertically transmitted *arsC*-like genes without any contribution to As resistance were found to exist in the 23 *Pantoea* strains. Besides these two *arsC*-like genes, As resistance gene clusters *arsRBC* or *arsRBCH* were found in 15 *Pantoea* genomes. These *ars* clusters were acquired by horizontal gene transfer (HGT) from sources related to *Franconibacter helveticus, Serratia marcescens*, and *Citrobacter freundii*. The insights gained in this study improve our understanding on the complex evolutionary history of As resistance genes and their roles in the biogeochemical cycling of As.

## Materials and Methods

### Phylogenetic Analysis

Phylogenetic trees of *Pantoea* species were constructed based on 100 single-copy core proteins shared by 23 *Pantoea* genomes and the genome of *Tatumella* sp. NML 06-3099 according to the following three methods: maximum likelihood (ML), neighbor joining (NJ), and Bayesian inference (BI). ML and NJ trees were computed by applying models with 1,000 bootstrap replicates and uniform rates in MEGA5 (Tamura, 2011). Multiple alignments of amino acid sequences were carried out by ClustalW, and the CONSEL program was used to select the best model of the trees (Shimodaira and Hasegawa, 2001; Thompson et al., 2002). The BI tree was generated using the MrBayes package with mixed models (Ronquist et al., 2012). The NJ tree of concatenated *arsRBC* homologs was generated according to the same method described above. MEGA5 or FigTree v.1.3.1^2^ was used to illustrate the constructed trees.

### Average Nucleotide Identity (ANI)

Assembled contigs were reconstituted from the RAST-generated GenBank files for 23 genomes by using the seqret function of the EMBOSS package (Rice et al., 2000). These 23 genomes were treated in the same manner to ensure that any biases were consistent across the entire dataset. JSpecies1.2.1 was used to analyze these contig sets for the ANI and tetramer usage patterns, using default parameters (Richter and Rosselló-Móra, 2009).

### Comparative Genomics

All of the orthologous pairs between *Pantoea* test genomes were identified by Pan Genome Analysis Pipeline (PGAP) (Zhao et al., 2012). The common dataset of shared genes among test strains was defined as their core genome. The total set of genes with test genomes was defined as the pan genome. The set of genes in each strain not shared with other strains was defined as the unique genes. The details of the strains used are listed in Supplementary Table S1.

### Construction of the Recombinant Plasmids and *Escherichia coli* Strains

A 3.86 kb *Bam*HI-*Xba*I DNA fragment containing the complete *ars1* cluster of *Pantoea stewartii* S301 (promoter region, 342 bp upstream of the start codon ATG of *arsR*, the contiguous four genes *arsR1B1C1H1* and 281 bp upstream of the start codon ATG of *arsH*) was PCR amplified with primers Ars1-F and Ars1-R (Supplementary Table S2). A 3.43 kb *Bam*HI-*Xba*I DNA fragment containing the complete *ars2* gene cluster of *P. agglomerans* Tx10 (a 280 bp region downstream of the stop codon TAA of *arsC2* and the contiguous ten genes *arsR2B2C2H2* and 328 bp downstream of the stop codon TAA of *arsH2*) was PCR amplified with primers Ars2-F and Ars2-R (Supplementary Table S2).

An 860 bp *Bam*HI-*Xba*I DNA fragment containing the complete *arsC1-*like gene of *P. stewartii* DC283 (promoter region, 221 bp upstream of the start codon ATG of *arsC1*-like gene, *arsC1*-like and 209 bp downstream of the stop codon TTA of *arsC1*-like gene) was PCR amplified with primers ArsC1-like-F and ArsC1-like-R (Supplementary Table S2). A 942 bp *Bam*HI-*Xba*I DNA fragment containing the complete *arsC2*-like gene of *P. stewartii* DC283 (promoter region, 265 bp upstream of the start codon ATG of *arsC2*-like gene, *arsC2*-like and 236 bp downstream of the stop codon TTA of *arsC2*-like gene) was PCR amplified with primers ArsC2-like-F and ArsC2-like-R (Supplementary Table S2).

The above PCR products were ligated to the *Bam*HI-*Xba*I site of plasmid pUC18, yielding plasmids pUC18-ars1, pUC18-ars2, pUC18-arsC1-like, and pUC18-arsC2-like. Then the plasmids were transferred to *E. coli* AW3110, yielding the recombinant *E. coli* AW3110-ars1, *E. coli* AW3110-ars2, *E. coli* AW3110-arsC1-like and *E. coli* AW3110-arsC2-like strains, respectively.

### Strains, Plasmids, and Culture Conditions

The strains and plasmids used in this work are summarized in Supplementary Table S3. *E. coli* and *Pantoea* strains were grown in LB medium (per liter contains: 10 g tryptone, 5 g yeast, and 10 g NaCl) or LB plates (LB medium with w/v 1.5% agar) at 30°C. When appropriate, antibiotics were added at the following concentration: 100 μg/mL ampicillin. Resistance to As species was tested by plating serial dilutions of cultures of each strain onto agar plates containing filtered sodium arsenate (Na_3_AsO_4_).

## Results

### Genomic Features

To date, 26 species have been reported in genus *Pantoea* and strains of nine species (*P. ananatis, P. agglomerans, P. stewartii, P. vagans, P. dispersa, P. septica, P. rodasii, P. rwandensis*, and *P. anthophila*) have been sequenced^3^. To study the genetic traits and phylogenetic history of As resistance in genus *Pantoea*, 23 strains were chosen, containing two to three standard strains sequenced in each species and five unidentified strains (Supplementary Table S1). A summary of features for these 23 sequenced genomes is listed in Supplementary Table S1. The G+C contents of the 23 genomes range from 53.4 to 59.1. These genomes vary in size by approximately 1.6 mega-bases in average (ranging from 4.02 to 5.68 Mb) with coding sequence (CDS) numbers ranging from 3580 to 8894, indicating a substantial strain-to-strain variation.

### Strain-Specific and Core Genes

To reveal the genomic features specific to each strain, we identified all orthologous pairs between the tested *Pantoea* genomes using PGAP. Our analysis of the total of 23 genomes revealed that a pan genome contains 48,207 putative protein-coding genes in the genus *Pantoea*. Out of these 48,207 genes, 10,896 (22.6%) were represented in the specific genomes of *Pantoea* spp., suggesting some frequency of horizontal gene acquisition from other taxa. The number of specific genes ranges from 131 to 1,285, with the smallest encoded by *P. vagans* C9-1 and the largest identified in *P. agglomerans* Tx10 (Supplementary Figure S1). The cluster of orthologous groups (COG) assignments reveal that a higher proportion of strain-specific genes in most of the strains can be assigned to the K (transcription), L (DNA replication), and M (cell wall/membrane/envelope biogenesis) categories (Supplementary Figure S2).

In contrast to the pan-genome, the core genome of *Pantoea* spp. contains 1,994 putative protein-coding genes, which represents 38.8–56.1% of the repertoire of protein coding genes of each strain, illustrating a small degree of genomic diversity in this group of bacteria (Supplementary Figure S1). The genomic analysis agrees with the fact that *Pantoea* strains are consistent in morphological and physiological appearance. Furthermore, the COG assignment results show that these core genes are in different functional categories (Supplementary Figure S3). In fact, the percentage of genes in each functional category remains rather similar (with an average divergence of 8.6%). This is consistent with an earlier report that larger prokaryotic genomes preferentially accumulate genes directly or indirectly involved in metabolism (Konstantinidis and Tiedje, 2004). These genes support a broader metabolic diversity, which, in turn, would improve the ecological success of *Pantoea* under more diverse environmental conditions.

### Phylogenic Analyses

**FIGURE 1:**
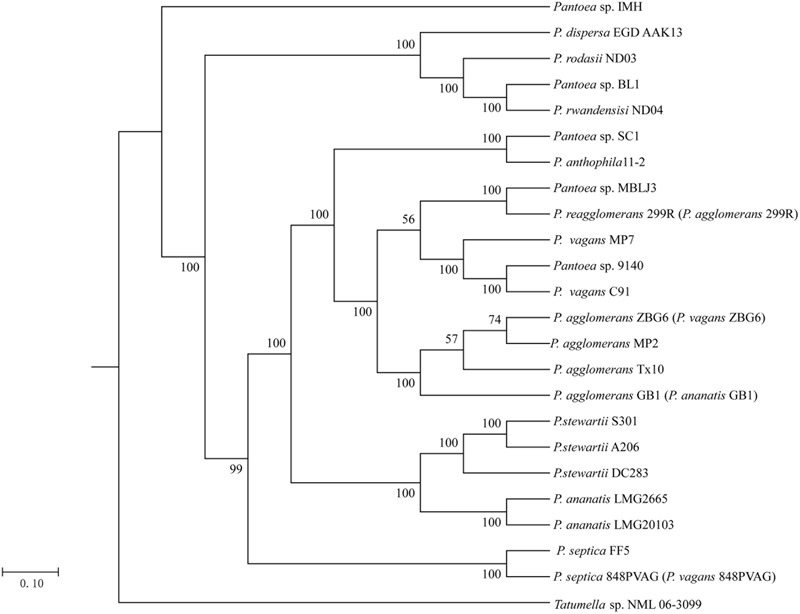
**Phylogenetic relationship of 23 *Pantoea* strains.** Maximum likelihood (ML) phylogenetic tree was constructed based on 100 single-copy core proteins shared by 23 genomes and an out-group *Tatumella* sp. NML 06-3099.

To associate the distribution of As resistance genes in *Pantoea* spp. with their phylogenetic affiliation, we constructed the phylogeny tree of the 23 *Pantoea* spp. based on 16S rRNA gene sequences using NJ methods rooted by *Tatumella* sp. NML 06-3099 (Supplementary Figure S9). This phylogenetic tree showed that the strains in the same species reported were grouped together except strain 848PVAG. At the same time, we constructed the phylogeny of the 23 genomes based on concatenation of the 100 core genes that are present as single copies in a genome using the ML method and rooted by *Tatumella* sp. NML 06-3099 (**Figure 1**). The phylogenetic trees, inferred using BI and NJ methods (Supplementary Figures S4, S5), were congruent with the ML phylogenetic tree. These trees show that some strains in different species reported were grouped together, such as, FF5 and 848PVAG, ZBG6, GB1, MP2, and Tx10. The phylogeny based on the 100 core genes that are present as single copies in a genome showed a good correlation with that of 16S rRNA gene sequences, except for three strains ZBG6, GB1, and 299R. These results suggested that there were mistakes in the classification of *Pantoea* spp.. Further identification of the phylogenetic status of these strains was carried out as follows.

**FIGURE 2:**
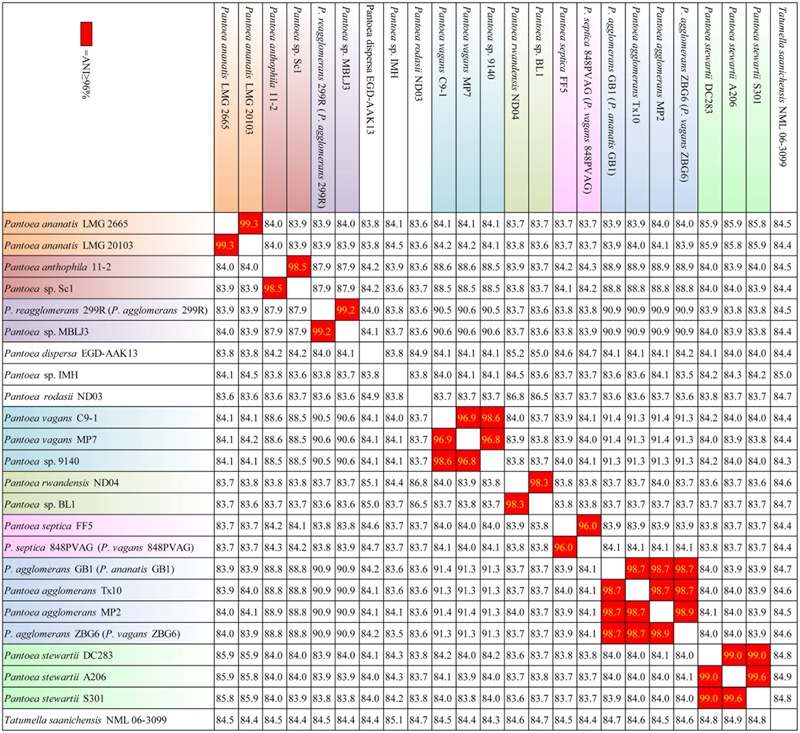
**Average Nucleotide Identity (ANI) (%) based on whole genome alignments.** ANI values are colored red according to historical species cutoff value (≥96%). Strains in one species are marked out the same color.

The information gained from the phylogenetic analysis provides an important depiction of the evolutionary relationship between different strains, but it does not translate directly into the overall similarity of the genomes, which is usually determined through the DNA-DNA hybridization (DDH). Herein, ANI approach was used to overcome the difficulty of conventional laboratory-based DDH in evaluating the genomic similarity of bacteria (Richter and Rosselló-Móra, 2009). The ANI results justified the conclusion of phylogenetic analysis. As shown in **Figure 2**, 23 strains were classified into 12 species based on their ANI ≥ 96%. For examples, LMG2665 and LMG20103 resulted in a higher ANI (99.3%), suggesting that they belong to the same species (*P. ananatis*). Strain 9140 and C91 resulted in a higher ANI (98.6%), suggesting that they belong to the same species (*P. vagans*). It was noteworthy that *Panotea* sp. IMH represented a novel species for the ANI ≥ 96% between IMH and other strains.

Strains MP2, Tx10, GB1, and ZBG6 which grouped together were identified as strains of *P. agglomerans*. Meanwhile, this result confirms the synonymy of *P.* FF5 and 848 PVAG (*P. vagans*), and suggests that 299R is not a member of species *P. agglomerans.* In agreement with the phylogenetic analysis, our ANI results indicate that there are mistakes in the classification of strain 299R, 848PVAG, GB1, and ZBG6. This mis-classification was also reported in other genus and generally corrected with the advance in technology (Goris et al., 2007). To associate the distribution of As-related genes with their phylogenetic affiliation, in this article below we renamed strain 299R to *P. reagglomerans* 299R (*P. agglomerans* 299R), 848PVAG to *P. septica* 848PVAG (*P. vagans* 848PVAG), GB1 to *P. agglomerans* GB1 (*P. ananatis* GB1), and ZBG6 to *P. agglomerans* ZBG6 (*P. vagans* ZBG6).

### Distribution and Organization of As-Related Genes in *Pantoea* Genomes

**FIGURE 3:**
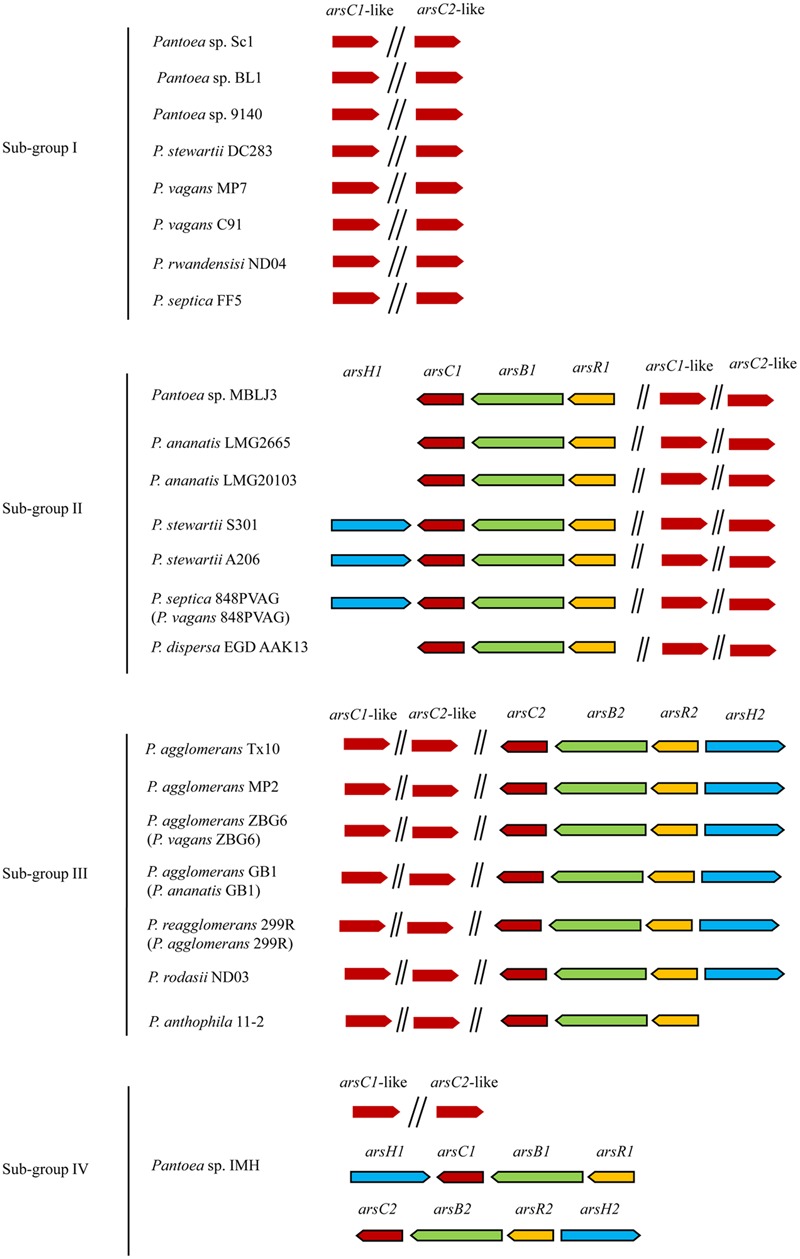
**Distribution and organization of *ars* genes and *arsC*-like genes in 23 *Pantoea* strains.** *arsC, arsB, arsR, arsH*, and *arsC*-like genes are marked with different colors. There are only *arsC*-like genes in Sub-group I, *arsR1B1C1* or *arsR1B1C1H1* in Sub-group II, *arsR2B2C2* or *arsR2B2C2H2* in Sub-group III, and both *arsR1B1C1H1* and *arsR2B2C2H2* in Sub-group IV.

Only As resistance genes (*ars* genes) including *arsR, arsB, arsC*, and *arsH* were detected in most *Pantoea* genomes (Supplementary Table S4 and **Figure 3**). The *arsC* gene encoding arsenate reductase is involved in the transformation of As(V) to As(III), which is then excreted by the As efflux pump ArsB encoded by the *arsB* gene. Nevertheless, *aio, arr*, and *arsM* were not found in *Pantoea* genomes, suggesting that cytoplasmic As(V) reduction and As(III) extrusion are the As resistance strategy used in genus *Pantoea* spp.. This mechanism benefits the bacteria itself, though it enhances the toxicity to the surrounding environment.

The *ars* genes in a genome are prone to group together as *ars* clusters (*arsRBC* and *arsRBCH*). Although comparison of the COG assignments of 23 genomes revealed that the DNA sequences between homologous genes within these *ars* clusters are conserved, some variations exist in DNA sequences, which can be divided into two sub-groups (*ars1* and *ars2*) (**Figure 3**). Unlike the two *ars* clusters in *Pantoea* sp. IMH, only one *ars* cluster, either *ars1* or *ars2*, was observed in other strains (Supplementary Table S2 and **Figure 3**). The *ars* gene clusters generally exhibited more than 80% identity within each sub-group and about 54% identity between two sub-groups. Actually, the *ars* clusters were not detected in eight strains including Sc1, BL1, 9140, DC283, MP7, C91, ND04, and FF5. Moreover, two *arsC*-like genes with only 25% homology (*arsC1*-like and *arsC2*-like) were found in the 23 genomes. Based on the different *ars* genes distributions, the 23 strains were categorized into four sub-groups and discussed as follows. The overall distribution and organization of As resistance genes in 23 *Pantoea* strains are summarized in **Figure 3**.

### Evolution and the Origin of *ars* Clusters

**FIGURE 4:**
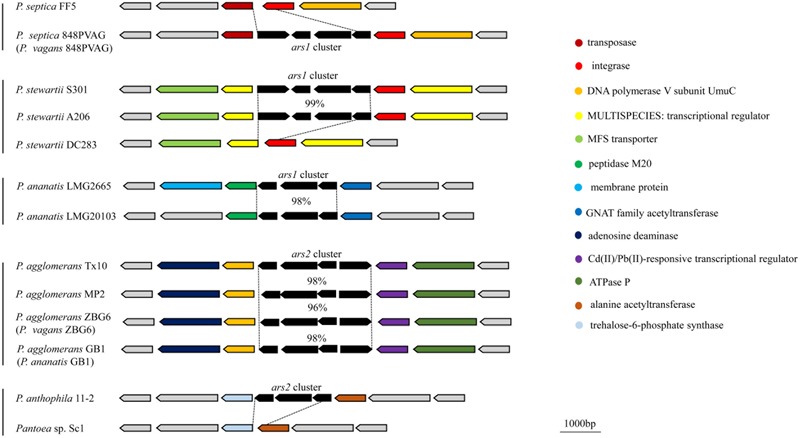
**Synteny of the chromosomal regions flanking the *ars* gene cluster among each species-group**.

The distribution and organization of *ars* genes in *Pantoea* raise a question as to their evolution. The deviant G+C content is used as a detect method of HGT (Ochman et al., 2000; Xie et al., 2014). We detected the G+C content of *ars* clusters and their corresponding genomes. The results showed that the G+C contents of the *ars1* clusters are higher than those of the genomes in *Pantoea* strains (56.3–57.8 vs. 53.4–54.7) except *P. septica* 848PVAG (*P. vagans* 848PVAG) and *P. dispersa* EGD-AAK13; the G+C contents of the *ars2* clusters are lower than those of the genomes in *Pantoea* strains (50.6–52.4 vs. 53.7–58.8), showing variation of G+C content between clusters and the corresponding genomes. These results indicated that these *ars* clusters may be acquired in *Pantoea* strains by HGT (Supplementary Table S4 and Figure S6). To further elucidate the evolution of the *ars* gene clusters, we compared the chromosomal regions flanking the *ars* gene clusters among the 23 *Pantoea* strains and found that the genes in the upstream and downstream regions were conserved among strains of the same species (**Figure 4**). For example, the DNA polymerase V subunit UmuC gene and adenosine deaminase gene in the upstream and Cd(II)/Pb(II)-responsive transcriptional regulator gene and ATPase P gene in the downstream are conserved for the *ars* clusters in the strain Tx10, MP2, ZBG6, and GB1 within the species *P. agglomerans* strains. The same species strains share the same insertion sites, whereas the different species’ strains result in different insertion sites, suggesting that *ars* clusters may be acquired more than once.

Interestingly, as shown in **Figure 4**, the flanking regions of the *ars* gene clusters in strain *P. stewartii* S301 and *P. stewartii* A206 were homologous to the corresponding regions of strain *P. stewartii* DC283; the same phenomenon was found in strain *P. septica* 848PVAG (*P. vagans* 848PVAG) and *P. septica* FF5, and strain *P. anthophila* 11-2 and *Pantoea* sp. Sc1. This result suggests that *ars* clusters may be lost in *P. stewartii* DC283, *P. septica* FF5, and *Pantoea* sp. Sc1.

**FIGURE 5:**
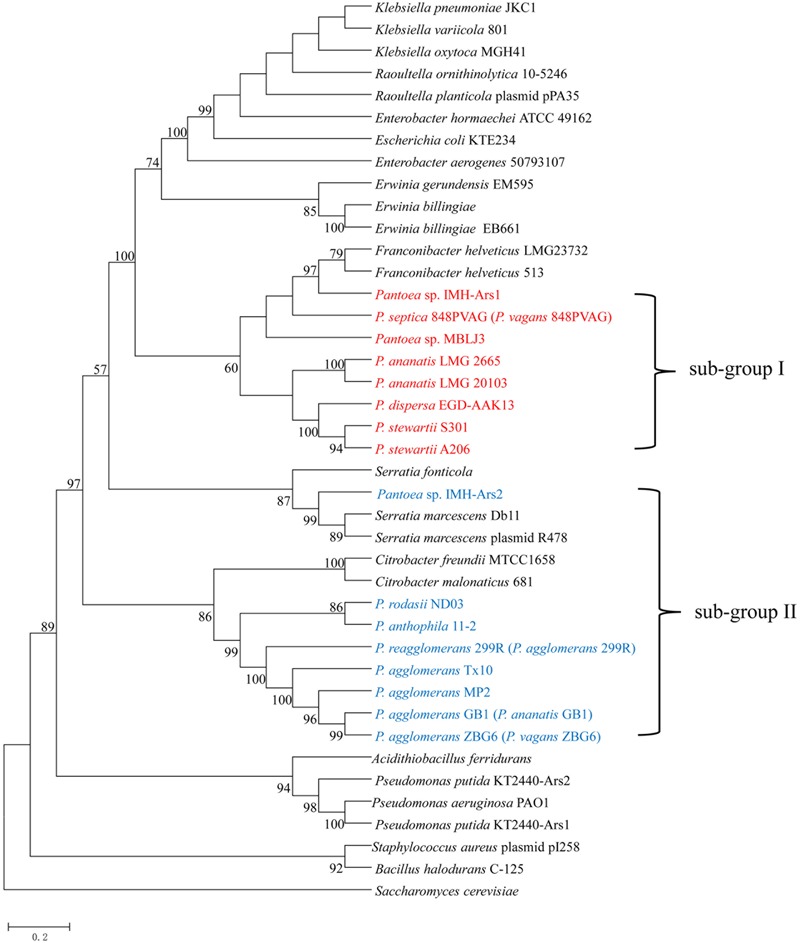
**Neighbor joining phylogenetic tree of the ArsRBC protein sequences derived from *Pantoea* spp. strains and other representative species.** A total of 1,000 bootstrap replicates was made, and bootstrap values are indicated at each node. *ars1* cluster grouped together marked in red, *ars2* cluster formed in another group marked in blue.

To gain insights into the origin of *ars* genes clusters in *Pantoea*, a NJ phylogenetic tree was constructed based on the ArsRBC protein sequences. As shown in **Figure 5**, the strains possessing *ars1* and *ars2* clusters form separate groups. Notably, the phylogeny reveals that the *ars1* and *ars* clusters of *F. helveticus* were sister groups, and *ars2* grouped to *ars* clusters of *S. marcescens* and *C. freundii*. These results imply that the *ars1* cluster may be acquired via HGT from *F. helveticus*, and *ars2* from *S. marcescens and C. freundii* in early evolutionary history.

### Two *arsC*-Like Genes in *Pantoea*

**FIGURE 6:**
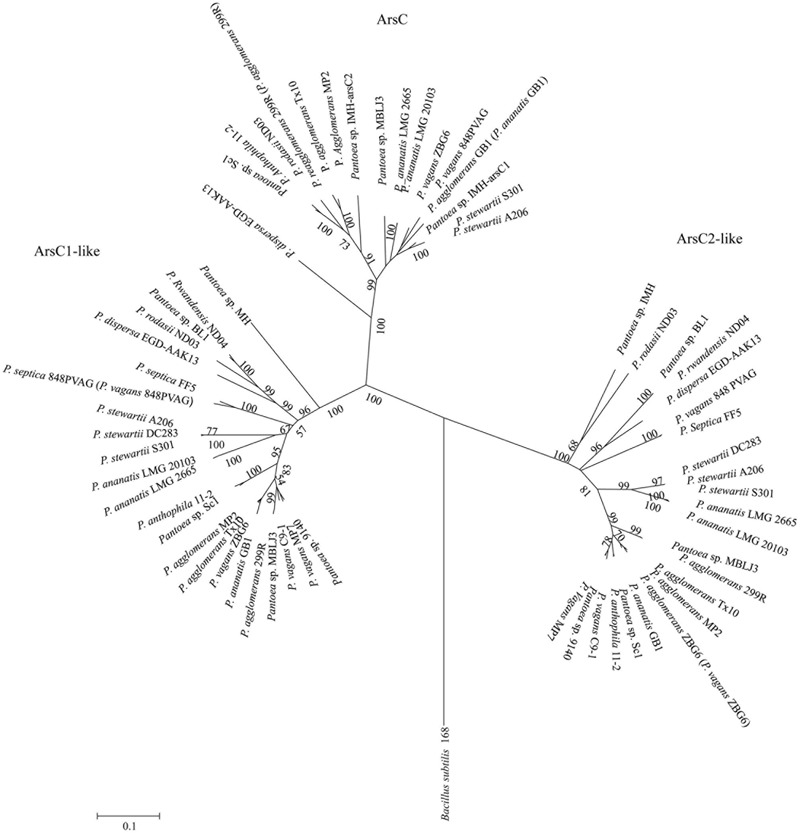
**Neighbor-joining tree based on ArsC/ArsC-like proteins.** ArsC/ArsC-like sequences were derived from 23 *Pantoea* strains and the representative microorganisms. ArsC sequence of *Bacillus subtilis* 168 was used as an out group.

Our studies reveal that two *arsC*-like genes (*arsC1*-like and *arsC2*-like) are found in the 23 genomes with just 25% homology (**Figure 3**). Our phylogenetic analysis showed that the ArsC-like sequences formed distinct groups, which were clearly divergent from conventional arsenate reductase (**Figure 6**). It was reported that Cys-12, Arg-60, Arg-94, and Arg-107 were four conserved residues of the ArsC protein in the process of arsenic resistance (Gladysheva et al., 1996). Cys-12 was identified as a catalytic residue and was activated by nearby residues Arg-60, Arg-94, and Arg-107 (Martin et al., 2001). Alignment analysis of a*rsC* and *arsC*-like genes shows that Cys-12 and Arg-94 residues were conserved, but residues Arg-107 and Arg-60 in two ArsC-like proteins were not conserved (Supplementary Figure S8). These results suggest that these two *arsC*-like genes are not involved in the As resistance.

To explore the evolution of these two *arsC*-like genes in *Pantoea*, molecular phylogenetic analysis, molecular conservation, and linear representation analysis were used (Rice and Lampson, 1995; Nelson et al., 1999; Brochier-Armanet and Forterre, 2006; Dagan et al., 2008). The comparative analysis showed that the two *arsC*-like genes are conserved in all of the 23 (Supplementary Figure S7). Phylogenetic analysis showed that *arsC1*-like and *arsC2*-like genes were grouped together, respectively (**Figure 6**). These results suggested that the two *arsC*-like genes evolved with a possible evolutionary scenario of that there is a common ancestor. Further, we compared the flanking regions of the two *arsC*-like genes. Interestingly, two genes in the upstream (the uracil phosphoribosyl transferase genes and uracil/xanthine transporter genes) and two genes in the downstream (sulfur reduction protein DsrE and GntR family transcriptional regulator genes) are conserved for *arsC1*-like genes. The two genes in the upstream (DNA-binding response regulator genes and multidrug efflux RND transporter permease genes) and two genes in the downstream (succinyl-diaminopimelate desuccinylase genes and membrane protein genes) are also conserved for *arsC2*-like genes (Supplementary Figure S7). This observation also suggests that *arsC1*-like and *arsC2*-like genes were the vertical genetic genes in the genus *Pantoea*. Possibility, they may have been the main As resistance contributors in early times and later had evolved with deviance.

### Functional Analysis of *ars* Gene and *arsC*-Like Genes

**FIGURE 7:**
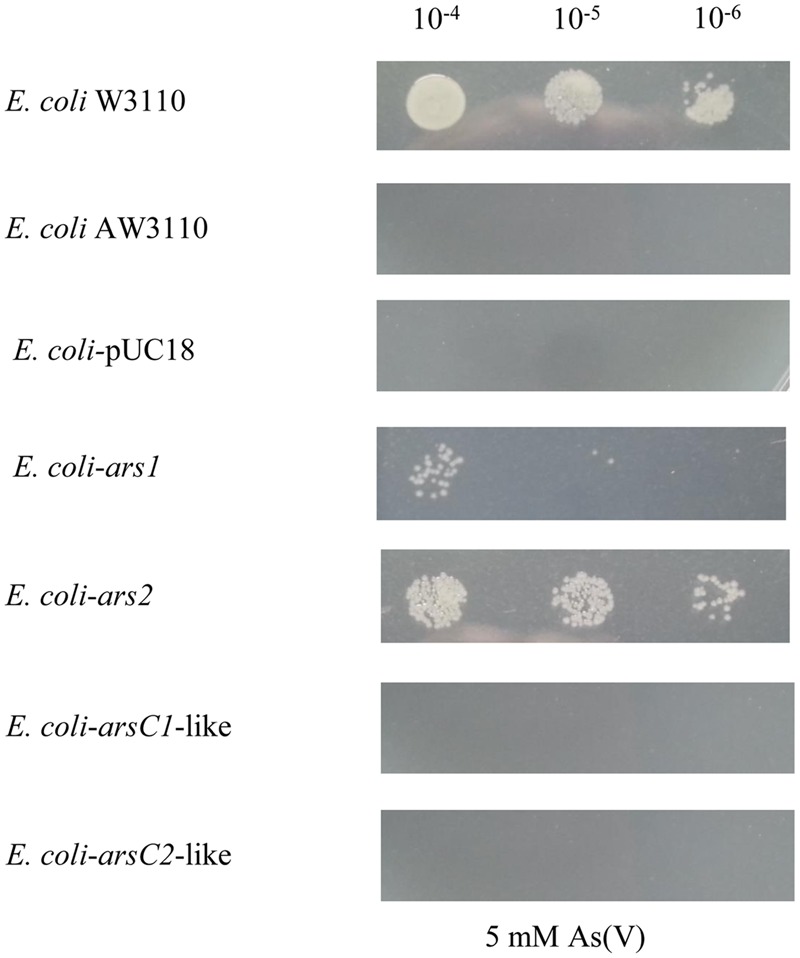
**The heterologous expression of *ars1, ars2, arsC1*-like and *arsC2*-like in *E. coli* AW3110.** The pUC18-*ars1*, pUC18-*ars2*, pUC18-*arsC1*-like and pUC18-*arsC2*-like plasmids (Supplementary Table S3) were transformed, along with the empty vector pUC18 in *Escherichia coli* AW3110. *E. coli* W3110 (with *arsRBC* cluster) as a control. Serial 5 μl dilutions of the strains were then plated on LB agar medium with 5 mM As(V). Pictures were taken after 48 h at 30°C.

To verify that the *ars* gene clusters are the contributors to the As resistance, the *ars1* cluster with its promoter from *P. stewartii* S301, a representative strain with the *ars1* cluster, and the *ars2* cluster with its promoter from *P. agglomerans* Tx10, a representative strain with the *ars2* cluster, were PCR amplified and then ligated into vector pUC18 and further transferred to *E. coli* AW3110 (without any As resistance genes). The growth of the yielded recombinant *E. coli* strains *E. coli-*ars1 and *E. coli-*ars2, was tested in 5 mM concentration As(V). As shown in **Figure 7**, both *E. coli-*ars1 and *E. coli-*ars2 survived in 5 mM As(V), and *E. coli-*ars1 grew better than *E. coli-*ars2. This result suggests that both the *ars1* and *ars2* clusters enabled *E. coli* AW3110 to resist As, and *ars1* seemed to have a more effective As resistance capability than *ars2*.

To test the functions of *arsC1*-like and *arsC2*-like genes, the *arsC1-*like gene and *arsC2*-like gene with their promoters from *P. stewartii* DC283 were PCR amplified and then ligated into vector pUC18 and further transferred to *E. coli* AW3110. As shown in **Figure 7**, neither the *arsC1*-like nor *arsC2-*like gene enables *E. coli* AW3110 to resist As. In line with the alignment result, the function analysis demonstrates that these two *arsC*-like genes do not contribute to As resistance.

## Discussion

*Pantoea* is a genus with 26 members identified by DDH, a gold standard for prokaryotic species identification. However, laboratory-based DDH results may be irreproducible, and vary depending on the reannealing temperature (Gevers et al., 2005). With the rapid development in technology and decline in sequencing cost, promising new measurements such as ANI are being developed to evaluate the genomic similarity of bacteria (Richter and Rosselló-Móra, 2009). In this study, we identified the 23 *Pantoea* spp. phylogenetic status using ANI, together with phylogenetic trees based on concatenated sequences of the 100 core genes (**Figures 1, 2**). Our results showed that strain 299R, 848PVAG, GB1, and ZBG6 were misnamed. We reclassified strain 299R to *P. reagglomerans* 299R (*P. agglomerans* 299R), 848PVAG to *P. septica* 848PVAG (*P. vagans* 848PVAG), GB1 to *P. agglomerans* GB1 (*P. ananatis* GB1), and ZBG6 to *P. agglomerans* ZBG6 (*P. vagans* ZBG6). Our study provided data from genus *Pantoea* with a complex and controversial taxonomy and demonstrated the accuracy of a bioinformatics approach, such as ANI, to identify new species and to correct erroneous identifications from previous studies.

A previous study suggested that the *ars* system is a widespread As resistance mechanism (Páezespino et al., 2009). *Pantoea* sp. IMH was found to resist As by means of *ars* clusters. *arsRBC* is located on the large universal *Pantoea* plasmids of four stains including *P. agglomerans* E325, *P. agglomerans* MP2, *P. eucalypti*αB, and *P. anthophila* Sc1 (Maayer et al., 2012). This information leads to the hypothesis that plasmids may be involved in the evolution of As resistance mechanism by *ars* genes in *Pantoea* spp. However, there are untouched questions such as what are the mechanisms of the other *Pantoea* spp. and what is the evolutionary history of genetic elements involved in the As resistance? To answer these questions, we collected the genome sequences of 23 strains in nine species in NCBI (*P. ananatis, P. agglomerans, P. stewartii, P. vagans, P. dispersa, P. septica, P. rodasii, P. rwandensis*, and *P. anthophila*). The sequencing results provided us with mass genomic information to detect the presence and the locations of As-related genes in *Pantoea* spp. Our study for the first time systematically analyzed the As resistance genes and revealed the As resistance traits in genus *Pantoea*. Our research provided the definitive evidence that that As resistance strategy in *Pantoea* spp. only involved the detoxification mechanism through *ars* clusters, not the respiratory reduction mechanism through *arr* clusters. This detoxification strategy was obtained by HGT. This conclusion can likely to be extended to most bacteria. We speculate that evolutionarily ancient microbes were exposed to As surroundings on ancient earth (Oremland et al., 2009). To overcome the As-induced selection pressure, microbes evolved *ars* genes in their genomes for survival by HGT. Therefore, *ars* has very early origins and represents a widespread As resistance mechanism.

Two scattered *arsC*-like genes exist in each genome of the 23 *Pantoea* strains, but they exhibited no functional As resistance. It is rare for *arsC*-like genes to show no As resistance capabilities (Butcher et al., 2000; Saltikov and Newman, 2003). Compared to functional protein ArsC, residues Arg-107 and Arg-60 of ArsC-like protein were variant (Supplementary Figure S8). We speculate that in early times, the ancestor of *Pantoea* spp. evolved the *arsC* gene to resist As, but later evolved with deviance during adaption to As-free niches, and thus retained non-functional *arsC*-like genes in some genomes.

The *ars* genes are abundant and tend to organize in typical *arsRBC* cluster structures (**Figure 3**). Apart from these operons, *arsRBCH* operons are widely observed. In genus *Pantoea*, these kinds of structures were anticipated, for these strains descended from a recent common ancestor. Our study suggests that *ars* clusters may be acquired by HGT from *F. helveticus, S. marcescens*, and *C. freundii* strains. This is consistent with recent literature showing that bacterial As resistance and transformation was a trait acquired via HGT, driven by adaptation to habitats containing As (Cai et al., 2009; Villegas-Torres et al., 2011). Interestingly, *ars* clusters are absent in some strains, suggesting that some microbes may have lost their As resistance genes during adaption to As-free niches. In addition, the number of As resistance genes in strains isolated from As-rich environments is much higher than in strains from other environments (Macur et al., 2004; Sutton et al., 2009). Compared to the evolutionary pattern of *ars* operons (Rosen, 1999), the evolution of As resistance genes (*ars* clusters) in *Pantoea* spp. involves a mix of HGT and loss, providing insight into the complex evolutionary history of As resistance.

## Author Contributions

LW and CJ conceived and designed the study. LW performed the laboratory work and data analysis. LW, JW, and CJ drafted the tables and figures, and prepared the main manuscript.

## Conflict of Interest Statement

The authors declare that the research was conducted in the absence of any commercial or financial relationships that could be construed as a potential conflict of interest.

## Data Accessibility

The NCBI accession numbers of 23 draft genome sequences of *Pantoea* are listed in Supplementary Table S1.

## Footnotes

1 http://www.bacterio.net/pantoea.html

2 http://tree.bio.ed.ac.uk/software/figtree/

3 http://www.ncbi.nlm.nih.gov/genome/?term=pantoea
