# Supplementary material for: Comparative Genomic Analysis Reveals Organization, Function and Evolution of ars Genes in Pantoea spp
Source: Front Microbiol. 2017 Mar 21;8:471. doi: 10.3389/fmicb.2017.00471 (PMC5360009; doi:10.3389/fmicb.2017.00471)
Supplement: Supplementary file 1 [file Data_Sheet_1.PDF]

Supporting Information

Comparative Genomic Analysis Reveals Organization, Function and Evolution of *ars* Genes in *Pantoea* spp.

Liying Wang[a,b], Jin Wang[c], Chuanyong Jing[a,b] *

[a]State Key Laboratory of Environmental Chemistry and Ecotoxicology, Research Center for Eco−Environmental Sciences, Chinese Academy of Sciences, Beijing 100085, China.

[b]University of Chinese Academy of Sciences, Beijing 100049, China

[c]Department of Municipal & Environmental Engineering, School of Civil Engineering, Beijing Jiaotong University, Beijing 100044, China

***Corresponding author:** Dr. Chuanyong Jing; Tel: +86 10 6284 9523;

E-mail: cyjing@rcees.ac.cn

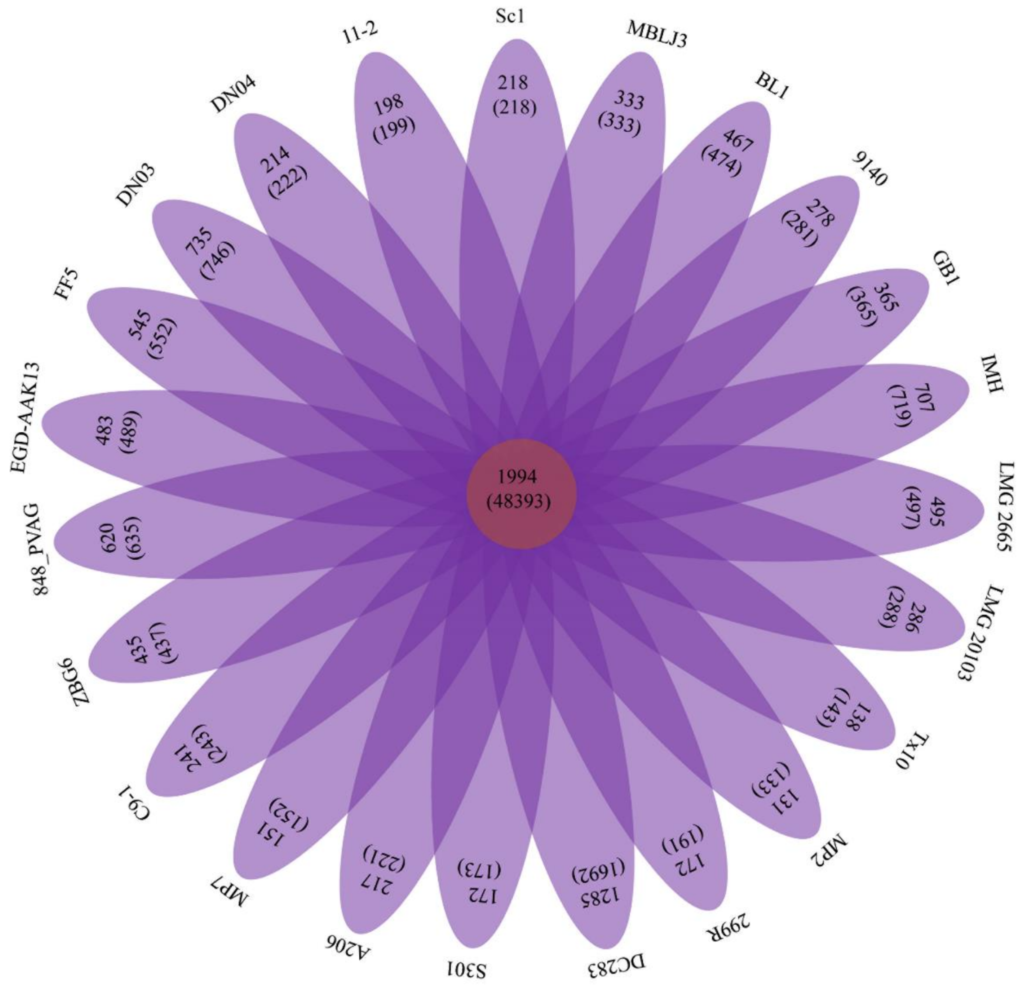

Figure S1. Genomic diversity of strains in the genus *Pantoea*. The number of orthologous coding sequences (CDSs) shared by all strains (i.e., the core genome) is in the center. Numbers in non-overlapping portions of each oval show the number of CDSs unique to each strain. The total number of protein coding genes within each genome is listed below the strain name.

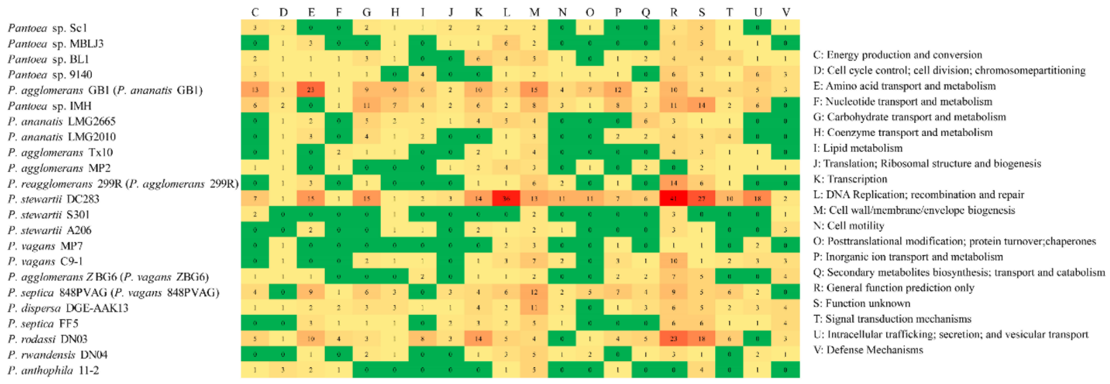

Figure S2. Functional classification of strain-specific genes in 23 *Pantoea* spp.. The number in each square represents the COG assignment in each functional category.

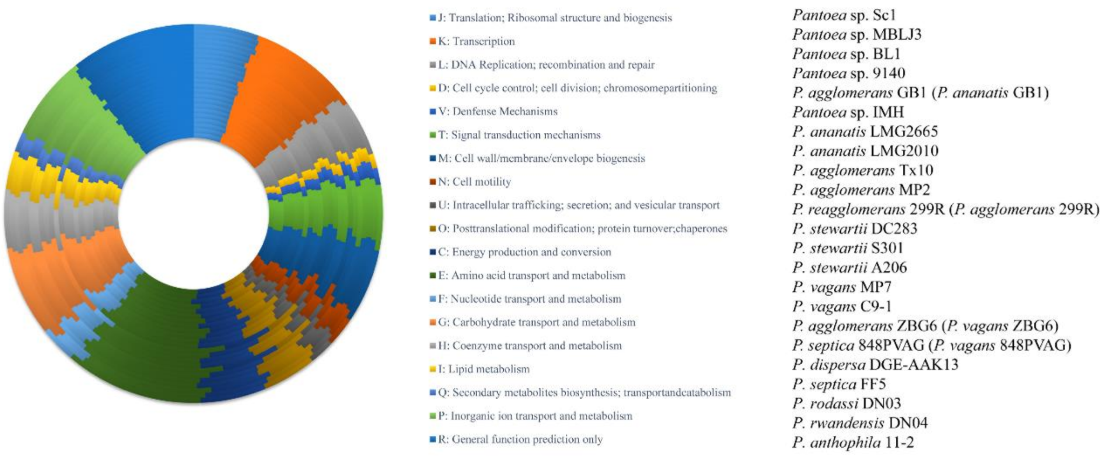

Figure S3. Function classification of Core genes in 23 *Panotea* strains. Taxa from inside of circle to outside of circle are from *Panotea* sp. Sc1 (top in the strain list) to *P. anthophila* 11-2 (down in the strain list).

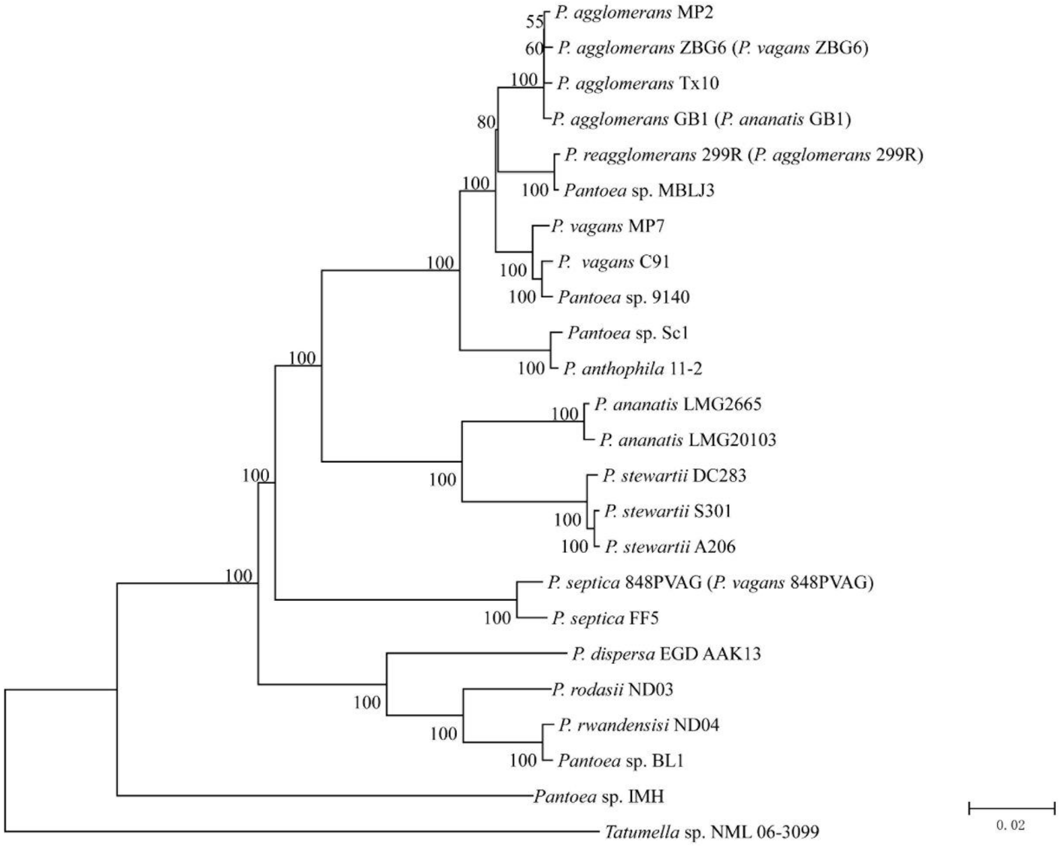

Figure S4. Phylogenetic relationship of 23 *Panotea* strains. Neighbor-joining phylogenetic tree was constructed based on 100 single-copy core proteins shared by 23 genomes and an out-group *Tatumella* sp. NML 06-3099.

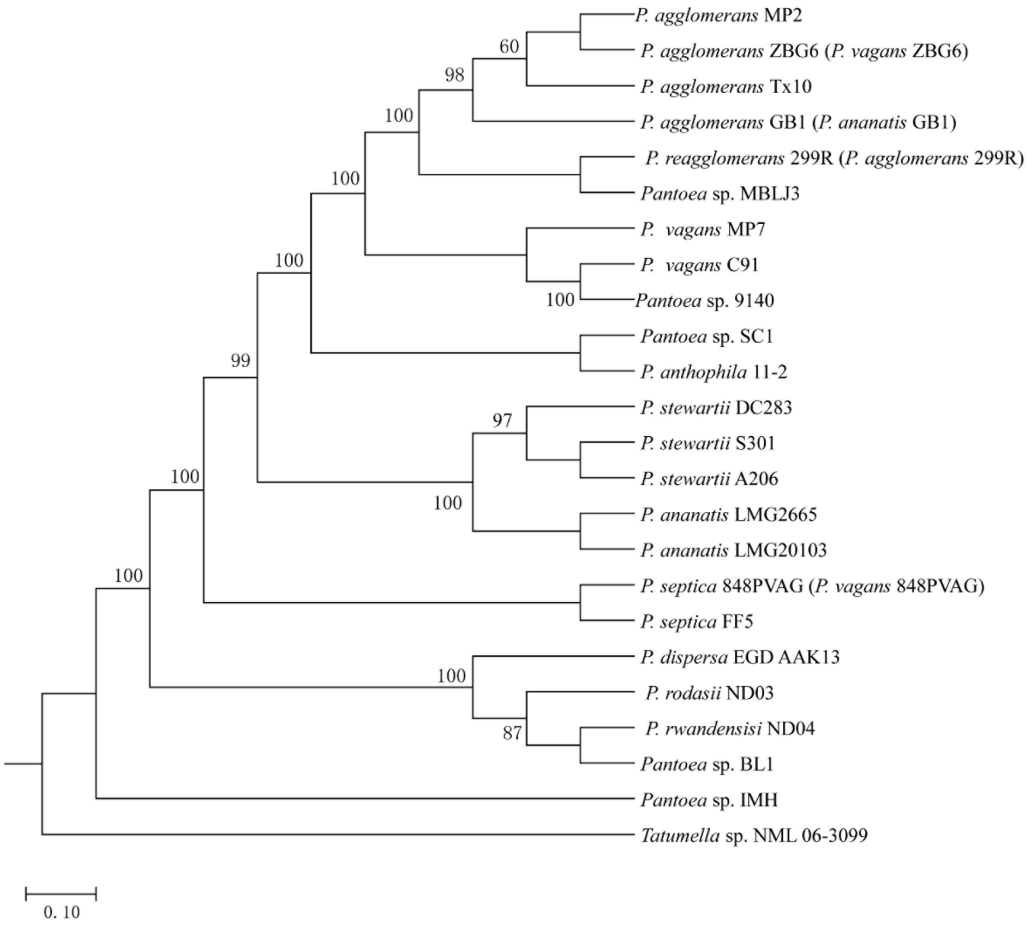

Figure S5. Phylogenetic relationship of 23 *Panotea* strains. Bayesian inferred phylogenetic tree was constructed based on 100 single-copy core proteins shared by 23 genomes and an out-group *Tatumella* sp. NML 06-3099.

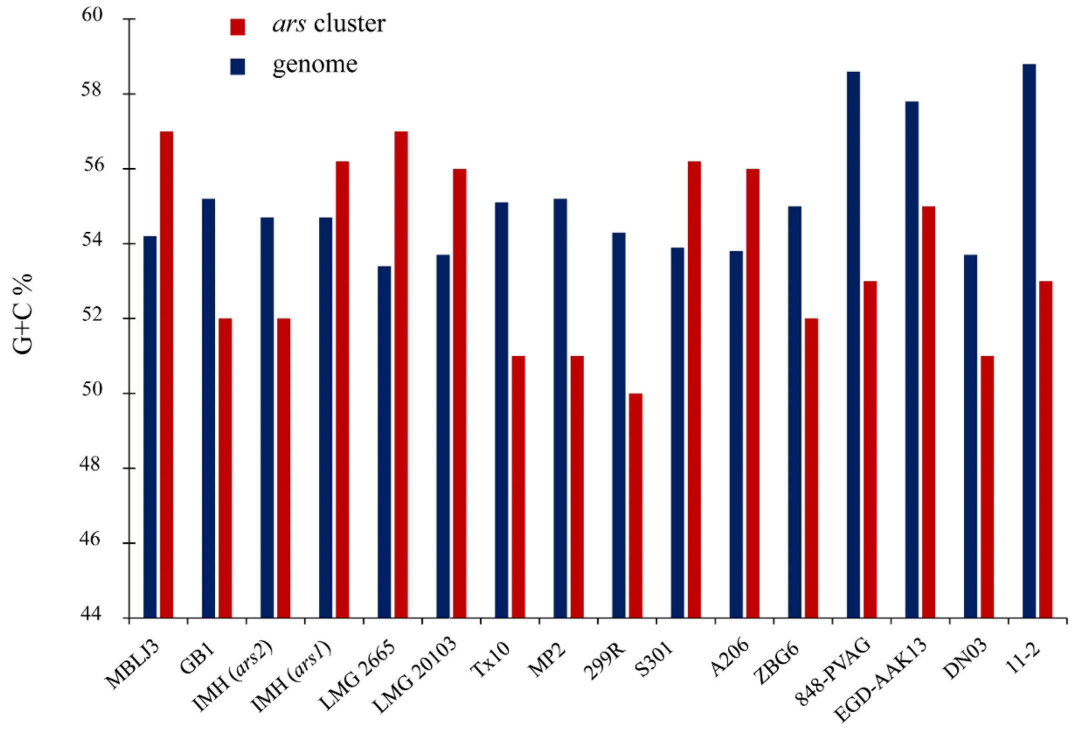

Figure S6. Comparison of G+C contents of the *ars* clusters with those of the average of the chromosomal genomes.

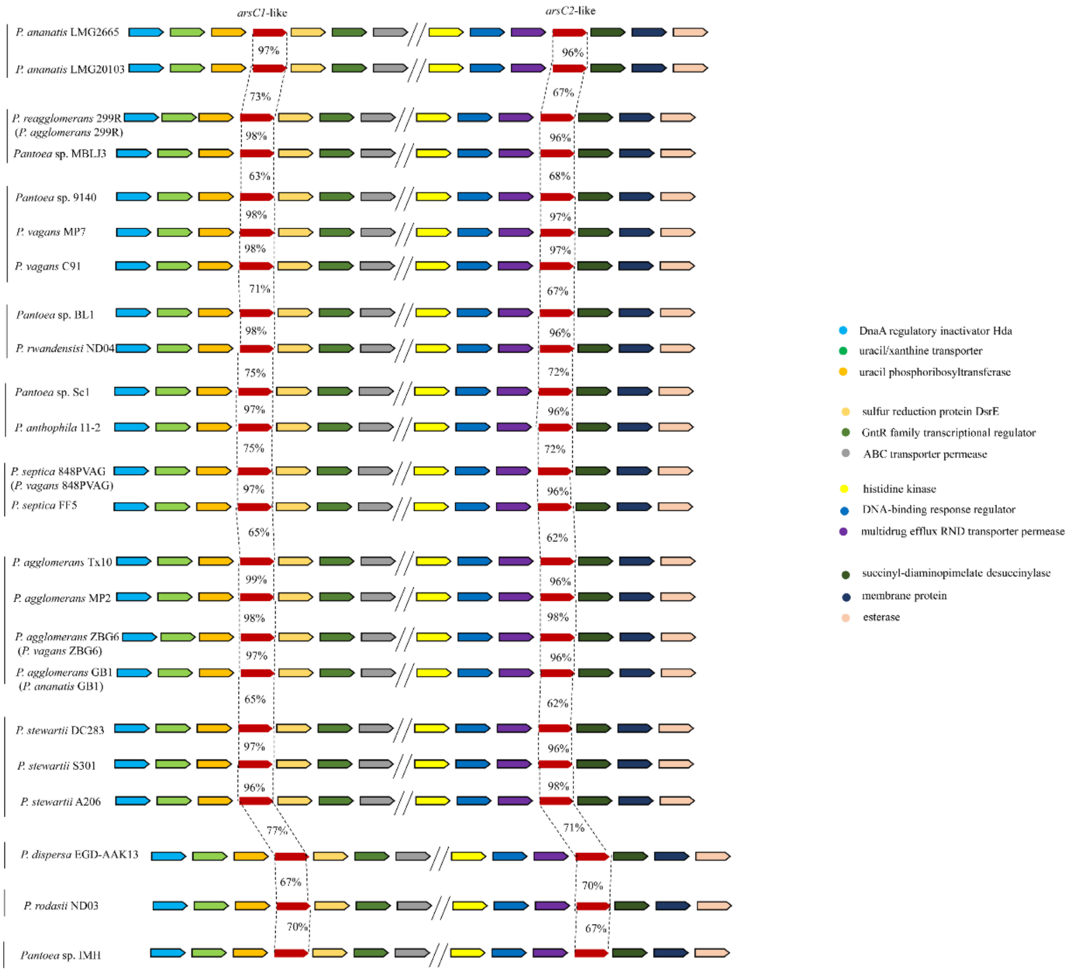

Figure S7. Synteny of the chromosomal regions flanking the two *ars*-like genes among the 23 *Pantoea* strains. The homology between the *arsC*-like genes were indicated below the genes.

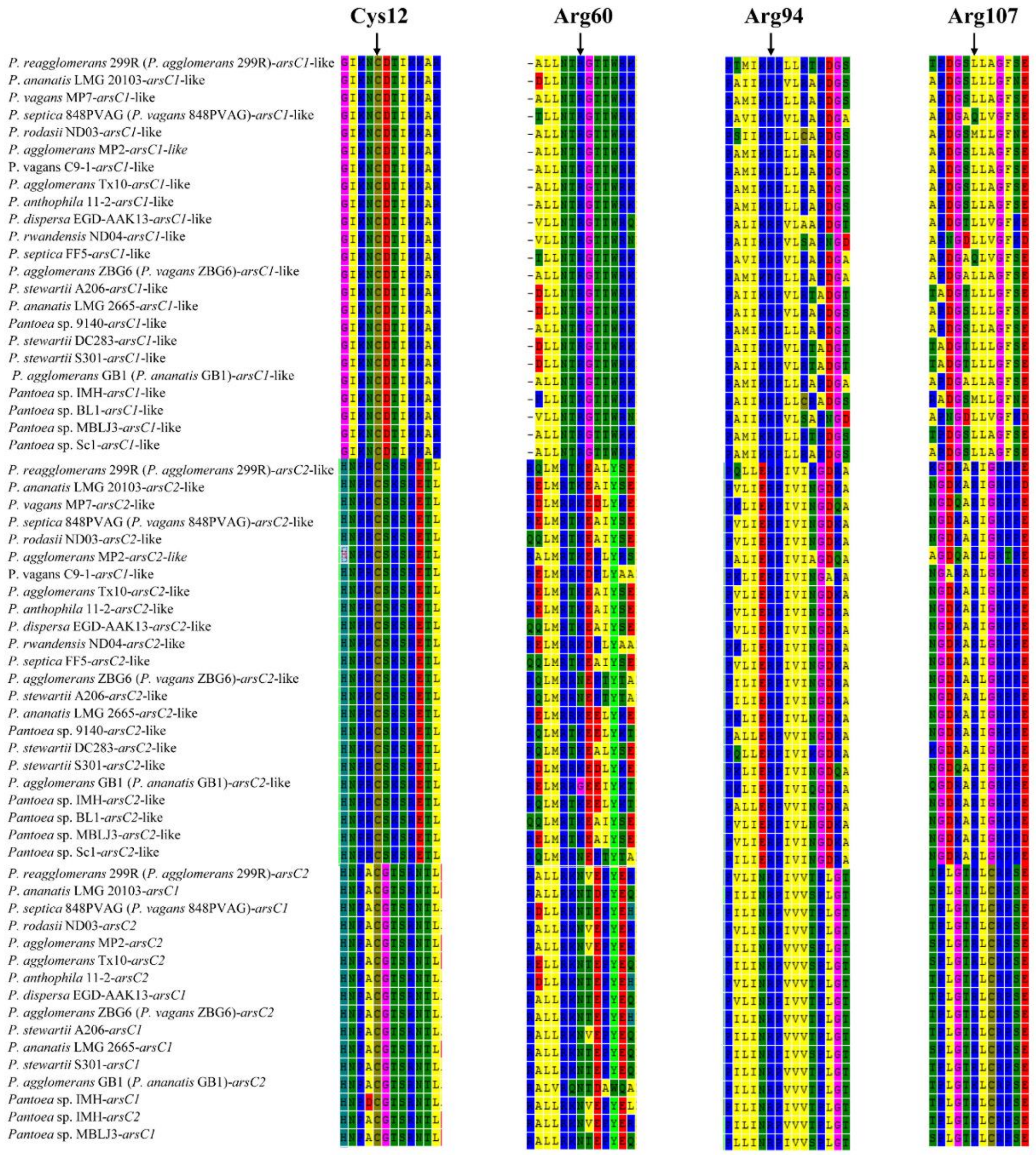

Figure S8. Alignment surrounding crucial residues Cys-12, Arg-60, Arg-94, and Arg-107 in ArsC and ArsC-like protein sequences from *Panotea* spp. Crucial residues Cys-12, Arg-60, Arg-94, and Arg-107 were indicated with vertical arrows.

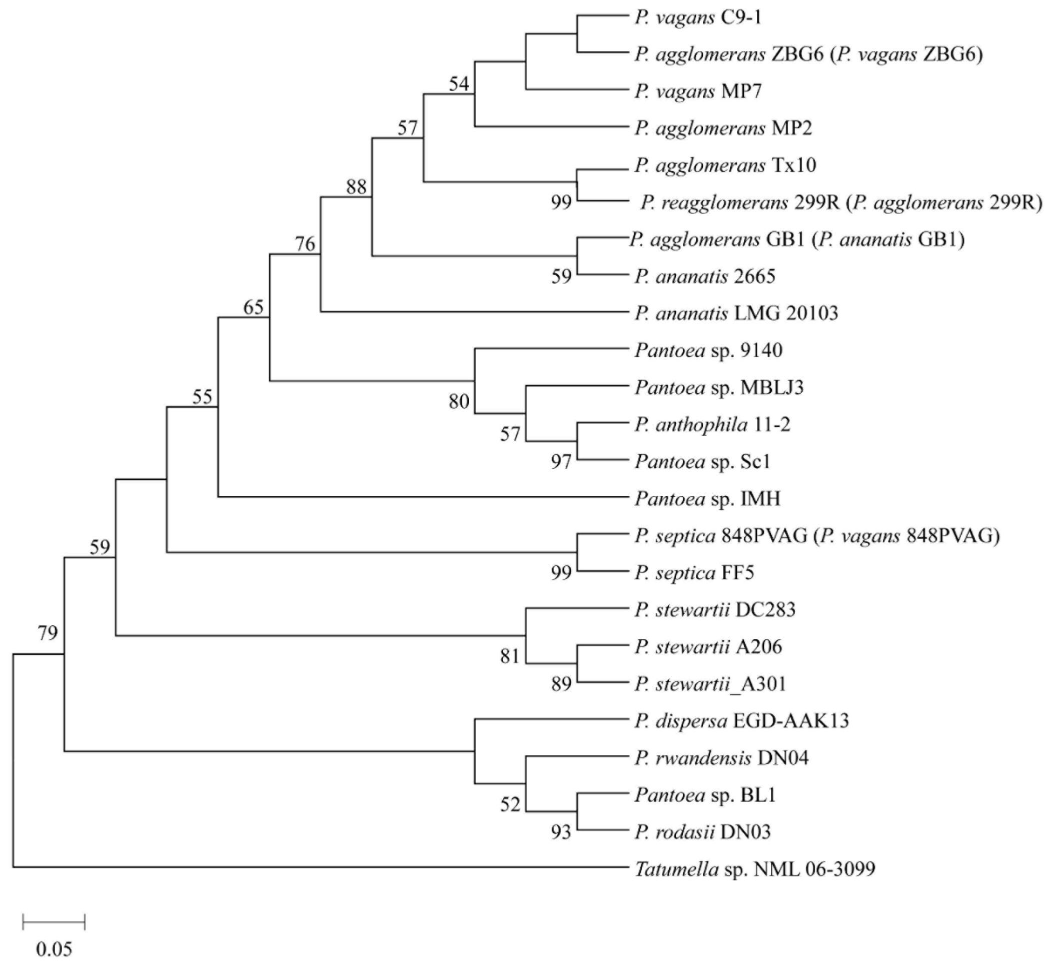

Figure S9. Phylogenetic relationship of 23 *Panotea* strains. Neighbor-joining phylogenetic tree was constructed based on 16S rRNA gene sequences of 23 *Pantoea* spp. and an out-group *Tatumella* sp. NML 06-3099.

Table S1 Genomic features of *Panotea* strains.

Strain	Status	Assembly level	Genome coverage	accession number	Genome size (Mb)	G+ C (%)	Proteins
Pantoea sp. Sc1	Draft	Contig	50 ×	AJFP00000000.1	4.45	57	4014
Pantoea sp. MBLJ3	Draft	Contig	236 ×	JSUT00000000.1	4.82	54.2	4394
Pantoea sp. BL1	Draft	Contig	61.95 ×	JZRH00000000.1	5.33	53.7	4812
Pantoea sp. 9140	Draft	Contig	unknown	JQNO00000000.1	4.84	55.3	4285
P. agglomerans GB1 (*P. ananatis* GB1)	Draft	Contig	51×	JYGW00000000.1	4.77	55.2	4158
Pantoea sp. IMH	Draft	Contig	100 ×	JFGT00000000.1	4.09	54.7	3609
P. ananatis LMG 2665	Draft	Scaffold	380 ×	JMJJ00000000.1	4.98	53.4	4444
P. ananatis LMG 20103	Complete	Complete	complete	CP001875.2	4.7	53.7	4104
P. agglomerans Tx10	Draft	Scaffold	347 ×	ASJI00000000.1	9.71	55.1	8894
P. agglomerans MP2	Draft	Scaffold	100 ×	JPKQ00000000.1	4.73	55.2	4287
P. reagglomerans 299R (*P. agglomerans* 299R)	Draft	Contig	268.4 ×	ANKX00000000.1	4.58	54.3	4162
P. stewartii DC283	Draft	Contig	19 ×	AHIE00000000.1	5.23	53.8	4822
P. stewartii S301	Draft	Scaffold	230 ×	LIIU00000000.1	4.49	53.9	4034
P. stewartii A206	Draft	Contig	168 ×	LIHC00000000.1	4.65	53.8	4211
P. vagans MP7	Draft	Scaffold	100 ×	JPKP00000000.1	4.6	55.3	4081
P. vagans C9-1	Complete	Complete	complete	CP002206.1	4.02	55.5	3580
P. agglomerans ZBG6 (*P. vagans* ZBG6)	Draft	Contig	133 ×	LFQL00000000.1	4.73	55	4249
P. septica 848PVAG (*P. vagans* 848-PVAG)	Draft	Contig	27 ×	JUQR00000000.1	4.99	58.6	4498
P. dispersa EGD-AAK13	Draft	Contig	28 ×	AVSS00000000.1	4.76	57.8	4299
P. septica FF5	Draft	Contig	unknown	CCAQ000000000.1	4.55	59.1	4121
P. rodasii DN03	Draft	Contig	29.2 ×	JTJJ00000000.1	5.68	53.7	5186
P. rwandensis DN04	Complete	Complete	complete	CP009454.1	4.33	53.9	3873
P. anthophila 11-2	Draft	Contig	50 ×	JXXL00000000.1	4.6	58.8	4142
Tatumella sp. NML 06-3099	Draft	Contig	34 ×	ATMI00000000.1	3.32	51.30	3123

Table S2 Primer used in this study.

Name	Sequence (5'-3')	Location/Target
Ars1-F	CGTTACCGGTGCATCCACAACTGTCATAACCCACTGAG	For cloning *ars1* cluster to pUC18
Ars1-R	GCATGGCGTCTCTAGAAGATATGATTTGTTTCCAGA	For cloning *ars1* cluster to pUC18
Ars2-F	CCATCATTACAAATTCTTGGCGCTGTGCGAGAAGTG	For cloning *ars2* cluster to pUC18
Ars2-R	CGACTATAGATGATCCAAGAGACCTGGAACGTTTGC	For cloning *ars2* cluster to pUC18
ArsC1-like-F	CGGTAGCCGGTGATCCTGGGATTGTCTCTCAGTACA	For cloning *arsC1*-like cluster to pUC18
ArsC1-like-R	GCAGCTCGACTCTAGACTGGCTGCACCAGGCCCAGAT	For cloning *arsC1*-like cluster to pUC18
ArsC2-like-F	CGGTATCCGCGAATCCATCATGATCAATATTGAAG	For cloning *arsC2*-like cluster to pUC18
ArsC2-like-R	GCATGTTGACACTAGATAGATGCGGTCAGCGTCCA	For cloning *arsC2*-like cluster to pUC18

Table S3 Strains and plasmids used in this study.

strains	characteristics	sources
E. coli W3110	K12 F^- IN (*rrnD-rrnE*)	Carlin *et al*., 1995
E. coli AW3110	K12 F^- Δ*ars*::*cam* F^- IN (*rrnD-rrnE*)	Carlin *et al*., 1995
**Plasmids**		
pUC18	cloning vector, Amp^R	TaKaRa
pUC18-*ars1*	pUC18 inserted with PCR fragment spanning complete *ars1* cluster with its native promoter，Amp^R	This study
pUC18-*ars2*	pUC18 inserted with PCR fragment spanning complete *ars2* cluster with its native promoter，Amp^R	This study
pUC18-*arsC1*-like	pUC18 inserted with PCR fragment spanning complete *arsC1*-like with its native promoter，Amp^R,	This study
pUC18-*arsC2*-like	pUC18 inserted with PCR fragment spanning complete *arsC2*-like with its native promoter，Amp^R	This study

Table S4 *ars* genes in the 23 genomes.

Strain	*ars* cluster gene	*arsC*-like gene	As resistance
Pantoea sp. Sc1	-	two *arsC*-like	No test
Pantoea sp. MBLJ3	*arsR1B1C1*	two *arsC*-like	No test
Pantoea sp. BL1	-	two *arsC*-like	No test
Pantoea sp. 9140	-	two *arsC*-like	No test
P. agglomerans GB1 (*P. ananatis* GB1)	*arsR1B1C1H1*	two *arsC*-like	No test
Pantoea sp. IMH	*arsR1B1C1H1, arsR2B2C2H2*	two *arsC*-like	Yes
P. ananatis LMG 2665	*arsR1B1C1*	two *arsC*-like	No test
P. ananatis LMG 20103	*arsR1B1C1*	two *arsC*-like	No test
P. agglomerans Tx10	*arsR2B2C2H2*	two *arsC*-like	No test
P. agglomerans MP2	*arsR2B2C2H2*	two *arsC*-like	No test
P. reagglomerans 299R (*P. agglomerans* 299R)	*arsR2B2C2H2*	two *arsC*-like	No test
P. stewartii DC283	-	two *arsC*-like	No test
P. stewartii S301	*arsR1B1C1H1*	two *arsC*-like	No test
P. stewartii A206	*arsR1B1C1H1*	two *arsC*-like	No test
P. vagans MP7	-	two *arsC*-like	No test
P. vagans C9-1	-	two *arsC*-like	No test
P. agglomerans ZBG6 (*P. vagans* ZBG6)	*arsR2B2C2H2*	two *arsC*-like	No test
P. septica 848PVAG (*P. vagans* 848-PVAG)	*arsR1B1C1H1*	two *arsC*-like	No test
P. dispersa EGD-AAK13	*arsR1B1C1*	two *arsC*-like	No test
P. septica FF5	-	two *arsC*-like	No test
P. rodasii DN03	*arsR2B2C2H2*	two *arsC*-like	No test
P. rwandensis DN04	-	two *arsC*-like	No test
P. anthophila 11-2	*arsR2B2C2*	two *arsC*-like	No test

note: - means without *ars* cluster

Reference:

Carlin, A., Shi, W., Dey, S. and Rosen, B. P. (1995). The *ars* operon of *Escherichia coli* confers arsenical and antimonial resistance. *J. Bacteriol.* 177, 981-986.
